# Lysophosphatidic acid species are associated with exacerbation in chronic obstructive pulmonary disease

**DOI:** 10.1186/s12890-021-01670-9

**Published:** 2021-09-23

**Authors:** Qingling Li, Weng Wong, Andrew Birnberg, Arindam Chakrabarti, Xiaoying Yang, David F. Choy, Julie Olsson, Erik Verschueren, Margaret Neighbors, Wendy Sandoval, Carrie M. Rosenberger, Michele A. Grimbaldeston, Gaik W. Tew

**Affiliations:** 1 Department of Microchemistry, Proteomics and Lipidomics, Genentech, Inc., South San Francisco, CA USA; 2 Department of Biomarker Discovery OMNI, Genentech, Inc., South San Francisco, CA USA; 3 Department of Biostatistics, Genentech, Inc., South San Francisco, CA USA; 4 Product Development Immunology, Infectious Disease and Ophthalmology, Genentech, Inc., South San Francisco, CA USA; 5 OMNI Biomarker Development, Genentech Inc., South San Francisco, CA USA

**Keywords:** Serum lysophosphatidic acid species, Lipidomic, Exacerbation, Chronic obstructive pulmonary disease

## Abstract

**Background:**

Chronic obstructive pulmonary disease (COPD) exacerbations are heterogenous and profoundly impact the disease trajectory. Bioactive lipid lysophosphatidic acid (LPA) has been implicated in airway inflammation but the significance of LPA in COPD exacerbation is not known. The aim of the study was to investigate the utility of serum LPA species (LPA16:0, 18:0, 18:1, 18:2, 20:4) as biomarkers of COPD exacerbation.

**Patients and methods:**

LPA species were measured in the baseline placebo sera of a COPD randomized controlled trial. Tertile levels of each LPA were used to assign patients into biomarker high, medium, and low subgroups. Exacerbation rate and risk were compared among the LPA subgroups.

**Results:**

The levels of LPA species were intercorrelated (rho 0.29–0.91). Patients with low and medium levels of LPA (LPA16:0, 20:4) had significantly higher exacerbation rate compared to the respective LPA-high patients [estimated rate per patient per year (95% CI)]: LPA16:0-low = 1.2 (0.8–1.9) (*p* = 0.019), LPA16:0-medium = 1.3 (0.8–2.0) (*p* = 0.013), LPA16:0-high = 0.5 (0.2–0.9); LPA20:4-low = 1.4 (0.9–2.1) (*p* = 0.0033), LPA20:4-medium = 1.2 (0.8–1.8) (*p* = 0.0089), LPA20:4-high = 0.4 (0.2–0.8). These patients also had earlier time to first exacerbation (hazard ratio (95% CI): LPA16:0-low = 2.6 (1.1–6.0) (*p* = 0.028), LPA16:0-medium = 2.7 (1.2–6.3) (*p* = 0.020); LPA20.4-low = 2.8 (1.2–6.6) (*p* = 0.017), LPA20:4-medium = 2.7 (1.2–6.4) (*p* = 0.021). Accordingly, these patients had a significant increased exacerbation risk compared to the respective LPA-high subgroups [odd ratio (95% CI)]: LPA16:0-low = 3.1 (1.1–8.8) (*p* = 0.030), LPA16:0-medium = 3.0 (1.1–8.3) (*p* = 0.031); LPA20:4-low = 3.8 (1.3–10.9) (*p* = 0.012), LPA20:4-medium = 3.3 (1.2–9.5) (*p* = 0.025). For the other LPA species (LPA18:0, 18:1, 18:2), the results were mixed; patients with low and medium levels of LPA18:0 and 18:2 had increased exacerbation rate, but only LPA18:0-low patients had significant increase in exacerbation risk and earlier time to first exacerbation compared to the LPA18:0-high subgroup.

**Conclusions:**

The study provided evidence of association between systemic LPA levels and exacerbation in COPD. Patients with low and medium levels of specific LPA species (LPA16:0, 20:4) had increased exacerbation rate, risk, and earlier time to first exacerbation. These non-invasive biomarkers may aid in identifying high risk patients with dysregulated LPA pathway to inform risk management and drug development.

**Supplementary Information:**

The online version contains supplementary material available at 10.1186/s12890-021-01670-9.

## Background

COPD is a chronic inflammatory disease in which airflow obstruction can result from emphysema and/or chronic bronchitis. Emphysema involves irreversible destruction of the alveoli, where greater emphysema is associated with increased exacerbation risk [1]. Chronic bronchitis involves airway wall thickening and mucus obstruction [2] which contribute to exacerbation risk and poorer outcomes [3, 4]. The frequency and severity of exacerbation have been shown to be associated with worse rate of lung function decline and health-related quality of life, where fewer than half of the patients survive for a further 5-years after a severe exacerbation [5].

COPD exacerbations are heterogenous events as the interactions between exacerbation triggers and host inflammatory responses are complex [6]. Accordingly, studies failed to identify consistent blood biomarkers associated with COPD exacerbation [7]. Conflicting results on the association of blood eosinophils with exacerbation risk, with either a positive [8–10] or no association [11, 12], highlight the heterogeneity of the patient population. High levels of fibrinogen are linked to increased risk of exacerbation and death in COPD [13–15], due to its association with emphysema [14], and its ability to identify patients with systemic inflammation together with other inflammatory biomarkers such as C-reactive protein and IL-6 [16]. These findings also underscore the significance of systemic perturbations that can occur in COPD patients.

Autotaxin-lysophosphatidic acid (ATX-LPA) pathway has been implicated in lung cancer and lung fibrosis [17, 18], but its role in COPD has not been established. ATX is a secreted glycoprotein and functions primarily as lysophospholipase D to remove the choline moiety from lysophosphatidylcholine (LPC), and generates majority of the bioactive lipid LPA detected in blood and inflamed tissues [19, 20]. LPA signals through G protein-coupled LPA receptors (LPAR_1–6_) expressed on many tissues and immune cells [21], to regulate in cell proliferation [22], fibrosis [23], and lymphocyte homing [18, 19]. LPA species vary in length and fatty acid saturation. The major forms of LPA increased in allergic inflammation are the 18:1, 20:4, 22:5 and 22:6 species [24]; in COPD, serum LPA 16:0 and 18:2 levels were positively correlated with FEV_1_%predicted in male COPD patients only [25], but the association of systemic LPA species with COPD exacerbation has not been reported.

As COPD clinical trials require large cohorts of patients to capture sufficient exacerbation events to observe the treatment benefits, biomarkers that identify high risk patients could help to enrich exacerbators and enable smaller or shorter duration clinical trials to accelerate drug development. In this study, we focused on the major LPA species (LPA16:0, 18:0, 18:1, 18:2, and 20:4) and assessed the relationship between systemic LPA levels with exacerbation frequency, risk and severity in COPD patients, with the aim of identifying non-invasive biomarkers that could inform clinical trial enrichment and patient risk management. Since different LPA species have been reported in asthma and COPD, information about the levels of individual LPA species may be more informative than the total LPA levels. Global lipidomic profiling was also performed to investigate the potential association of lipid changes with LPA and exacerbation.

## Patients and methods

### Patient cohort

Baseline serum samples from the placebo arm (n = 136) of a global COPD randomized controlled trial (NCT02546700) were used for LPA and lipid measurements. The study included GOLD stage II to IV patients, with a history of at least one exacerbation in the past 12 months and a smoking history of at least 10 pack-years. Patients with a current diagnosis of asthma were excluded. Clinical measures were collected at baseline and every 4 to 12 weeks thereafter during a 24-week placebo-controlled period. Chronic bronchitis was defined using St. George’s Respiratory Questionnaire COPD (SGRQ-C) cough and phlegm questions: patients were categorized as having chronic bronchitis (CB-SGRQ) if the cough was “most days a week” or “several days a week” and phlegm was “most days a week” or “several days a week”. An exacerbation was defined in the study protocol as new or increased COPD symptoms (e.g. dyspnea, sputum volume, and sputum purulence) for at least 2 consecutive days that led to treatment with systemic corticosteroids and/or antibiotics, or hospitalization. Exacerbation duration corresponded to the number of days patients were on systemic corticosteroids and/or antibiotics. Complete blood cell counts were measured by routine clinical laboratory tests. Serum IgE levels were measured on ImmunoCap® (Viracor Eurofins, MO). Plasma was collected in sodium citrate tubes for fibrinogen measurement using the Clauss method (Siemen BCS, Covance, NJ).

### Mass spectrometry LPA assays

LPA species were extracted using acidified butanol solvent as previously described [26]. Briefly, 10 µl of serum from each sample was pooled together as quality control samples. 500 µl disodium phosphate buffer (30 mM citric acid and 40 mM disodium phosphate, pH 4.0) and 2 ml butanol were added to 20 µl serum to extract lipids. The extracted samples were reconstituted in methanol and analyzed by liquid chromatography-mass spectrometry (LC–MS/MS). LC coupling to a QTRAP mass spectrometer was employed under negative ionization mode. HPLC separation of LPA was optimized on a C18 column to separate LPA from other lipids. Turbo-ion-spray source of mass spectrometer was set at 300 °C, N2 nebulization at 16 psi, N_2_ heater gas at 10 psi, curtain gas at 35 psi, collision-activated dissociation gas pressure was held at medium, turbo ion-spray voltage at −4500 V, declustering potential at −70 V, entrance potential at −10 V, collision energy at −28 V and collision cell exit potential at −10 V. Sample analysis was performed in multiple reactions monitoring mode. LPA species were monitored using the transitions [M–H]^−^ to 153.0. LPA species were shown to be stable within the experimental time frame, including storage at -80 °C freezer over a month and at 15 °C auto sampler over 55 h.

### Lipidomic profiling

Lipidomic measurement was carried out using a modified method derived from a previous study [27]. Patients with sufficient remaining serum volume (n = 134) were used for lipidomic profiling. Briefly, lipids were purified using dichloromethane, methanol and water two phases extraction. After direct infusion, lipid species were analyzed on a SelexION enabled 6500 QTRAP mass spectrometry (Sciex, Redwood City, CA) in multiple reaction monitoring mode. Lipid species were identified and quantified on the basis of characteristic mass spectrometry transitions.

### Statistical analysis

Statistical analyses were performed using R (version 3.5.3). LPA and lipid concentrations were log2 transformed when appropriate. The relationship between LPA levels, baseline demographics and other biomarkers were assessed using univariate linear regression or Spearman’s rank order method. Sex-specific tertile levels of each LPA were used to assign patients into biomarker high (highest tertile), medium, and low (lowest tertile) subgroups. Comparisons among the subgroups were assessed using ANOVA with Tukey HSD test, Student t-test or Kruskal–Wallis, for continuous measures; and Fisher’s Exact test for categorical measures. Logistic regression and Quasi-Poisson model were used to estimate exacerbation risk and rate, respectively. Cox proportional hazards regression was used to compare the time to first exacerbation. Covariates in exacerbation models included sex and stratification factors pre-specified in the study protocol: exacerbation history, smoking status (former versus current based on questionnaire), geographical region (American Continent versus rest of the world), COPD medications (double therapy: inhaler corticosteroids (ICS) and one bronchodilator versus triple therapy: ICS and two bronchodilators). *P*-value < 0.05 was considered as statistically significant. Lipid species detectable in at least 90% of patients were included in the analyses. Lipid concentrations were compared among LPA subgroups, using Kruskal–Wallis test followed by Benjamini–Hochberg correction (FDR) for multiple comparisons. FDR < 0.05 was considered as statistically significant.

## Results

### Baseline characteristics

Baseline characteristics of the patients were shown in Table 1. A higher proportion of men had more severe disease with 24% of men classified as GOLD stage 4 compared to 7% of women (*p* = 0.011). LPA concentrations, except for LPA20:4, were significantly lower in men compared to women (Additional file 1: Figure S1). LPA concentrations were not significantly different by smoking status or COPD medications (Additional file 1: Figure S1). There was no significant association between LPA levels with age, body mass index (BMI), FEV_1_ (absolute or %predicted), or FVC (not shown).

**Table 1:** Patient baseline characteristics

	Male N = 82	Female N = 54	*p *value
Age (years)	65.2 (6.8)	63.6 (8.1)	0.24
Race			
White, %	77(94)	50 (93)	
Black or African American, %	3 (4)	2 (4)	0.84
Others, %	2 (2)	2 (4)	
Body mass index	27.2 (4.9)	27.2 (5.5)	0.98
Former smokers, %	50 (61)	26 (48)	0.16
Post-bronchodilator FEV_1_			
Absolute (L)	1.4 (0.5)	1.2 (0.4)	0.0036
% predicted	43.8 (15.5)	51.3 (13.6)	0.0043
Post-bronchodilator FEV_1_/FVC ratio	0.45 (0.1)	0.52 (0.1)	0.00024
SGRQ-C score	57.9 (17.3)	57.1 (18.3)	0.80
GOLD, %			
Stage II	31 (38)	32 (59)	
Stage III	31 (38)	18 (33)	0.011
Stage IV	20 (24)	4 (7)	
Patients with bronchodilator reversibility, %	14 (17)	6 (11)	0.46
Patients with chronic bronchitis, %	67 (82)	41 (76)	0.52
Patients with severe exacerbation in previous 12 months, %	26 (32)	11 (20)	0.17
Median blood eosinophil count (cells per μL)	180 (130)	175 (183)	0.89
Median fibrinogen (g/L)	3.8 (1.4)	3.5 (0.9)	0.037

Data are n (%), mean (SD), or median (IQR). FEV_1_, forced expiratory volume in 1 s; FVC, forced vital capacity; SGRQ-C, St. George’s Respiratory Questionnaire COPD

**Fig. 1:**
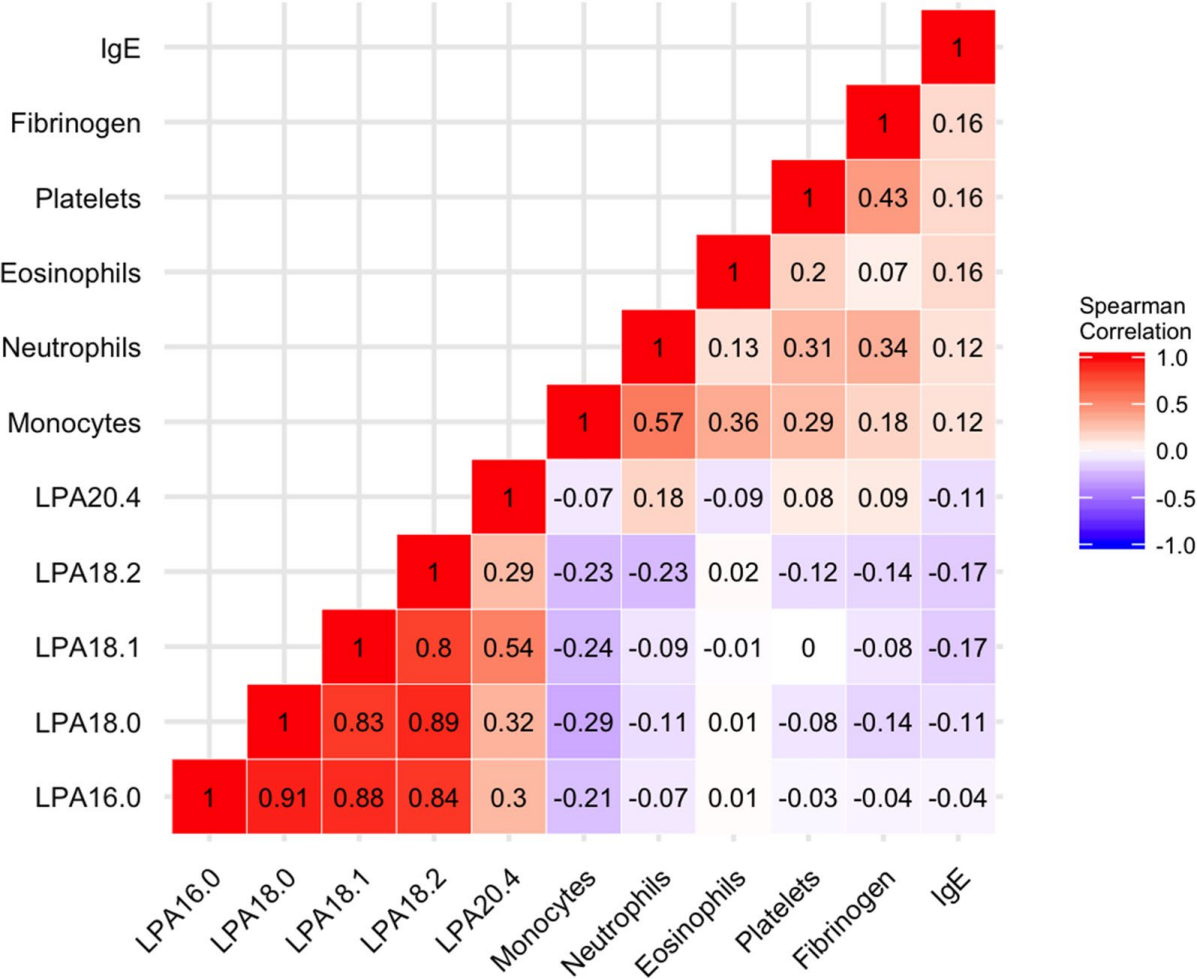
Baseline correlation of LPA species with biomarkers. Spearman’s rho values shown

The levels of LPAs (16:0, 18:0, 18:1 and 18:2) were highly correlated with each other (rho 0.80–0.91), but exhibited modest correlation with LPA20:4 (rho 0.29–0.54) (Fig. 1). LPA levels had no significant correlation with blood eosinophils, platelets, plasma fibrinogen or serum IgE.

Since LPA levels were significantly different between male and female patients, sex-specific tertile levels of each LPA were used to categorize patients into biomarker high, medium, and low subgroups. The tertile cutoff concentrations were shown in the respective LPA patient characteristic tables (Table 2, Additional file 1: Tables S1–S4). There were no significant differences in baseline characteristic among the LPA subgroups (Table 2, Additional file 1: Tables S1–S4) except for LPA16:0, where LPA16:0-low patients were younger compared to the LPA16:0-medium and -high subgroups (*p* = 0.00052), and LPA16:0-medium subgroup had lower BMI compared to the LPA16:0-high subgroups (*p* = 0.0069) (Table 2).

**Table 2:** Patient baseline characteristics by LPA16:0 subgroups

LPA16:0	Low N = 45	Medium N = 46	High N = 45	*p *value
LPA16:0 tertile concentration (µM)				
Male	< 0.12	0.12 to < 0.20	≥ 0.20	
Female	< 0.18	0.18 to < 0.30	≥ 0.30	
Age (years)	61.3 (7.6)	67 (6.6)*	65.3 (6.6)*	0.00052
Body mass index	27.6 (5.3)	25.3 (4.7)	28.5 (5)^+^	0.0069
Former smokers, %	24 (53)	29 (63)	23 (51)	0.47
Post-bronchodilator FEV_1_				
Absolute (L)	1.3 (0.5)	1.3 (0.5)	1.3 (0.4)	0.56
% predicted	46.1 (17.2)	46.4 (15.8)	47.5 (12.6)	0.90
Post-bronchodilator FEV_1_/FVC ratio	0.5 (0.1)	0.5 (0.1)	0.5 (0.1)	0.83
SGRQ-C score	58.8 (17)	55.7 (16.1)	58.2 (20)	0.68
GOLD, %				
Stage II	20 (44.4)	20 (43.5)	22 (48.9)	
Stage III	13 (28.9)	18 (39.1)	18 (40.0)	0.40
Stage IV	12 (26.7)	8 (17.4)	5 (11.1)	
Patients with chronic bronchitis, %	39 (86.7)	34 (73.9)	34 (75.6)	0.17
Patients with severe exacerbation in previous 12 months, %	13 (28.9)	12 (26.1)	12 (26.7)	0.97
Median blood eosinophil count (cells/μL)	185 (105)	160 (97.5)	200 (265)	0.15
Median fibrinogen (g/L)	3.5 (1.1)	3.6 (1.5)	3.5 (1)	0.26

Data are n (%), mean (SD), or median (IQR).

*Tukey test *p* < 0.05 compared to LPA-low

^+^Tukey test *p* < 0.05 compared to LPA-medium. FEV_1_, forced expiratory volume in 1 s; FVC, forced vital capacity; SGRQ-C, St. George’s Respiratory Questionnaire COPD

### LPA and exacerbation

**Fig. 2:**
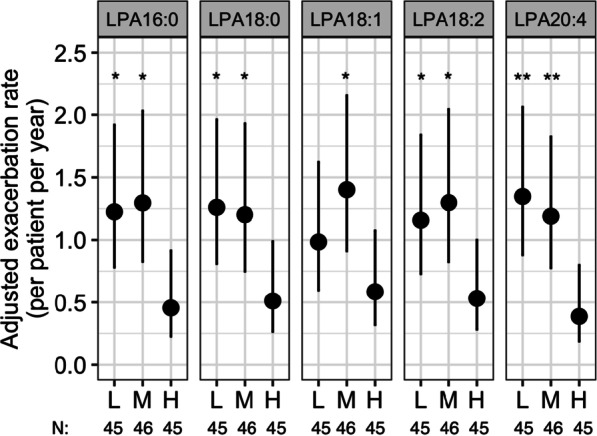
Rate of exacerbation over 24 weeks by baseline LPA profile. Adjusted exacerbation rates are estimates from a Quasi-Poisson regression model adjusted for the following covariates in addition to log(patient-years) as an offset: number of exacerbations within the last 12 months, smoking status, geographical region, baseline COPD medications, and sex. L = lowest-; M = medium-; H = highest-tertile of the respective LPA levels. P-values compared H to L or M subgroup. *p < 0.05; **p < 0.01. N, number of patients

Except for LPA18:1, exacerbation rate was significantly higher in patients with low and medium LPAs (LPA16:0, 18:0, 18:2 or 20:4) as compared to the respective LPA-high patients: (estimated rate per patient per year (95% CI)) LPA16:0-low = 1.2 (0.8–1.9) (*p* = 0.019), LPA16:0-medium = 1.3 (0.8–2.0) (*p* = 0.013), LPA16:0-high = 0.5 (0.2–0.9); LPA18:0-low = 1.2 (0.8–2.0) (*p* = 0.025), LPA18:0-medium = 1.2 (0.8–1.9) (*p* = 0.040), LPA18:0-high = 0.5 (0.3–1.0); LPA18:2-low = 1.2 (0.7–1.8) (*p* = 0.049), LPA18:2-medium = 1.3 (0.8–2.0) (*p* = 0.024), LPA18:2-high = 0.5 (0.3–1.0); LPA20:4-low = 1.4 (0.9–2.1) (*p* = 0.0033), LPA20:4-medium = 1.2 (0.8–1.8) (*p* = 0.0089), and LPA20:4-high = 0.4 (0.2–0.8) (Fig. 2).

**Fig. 3:**
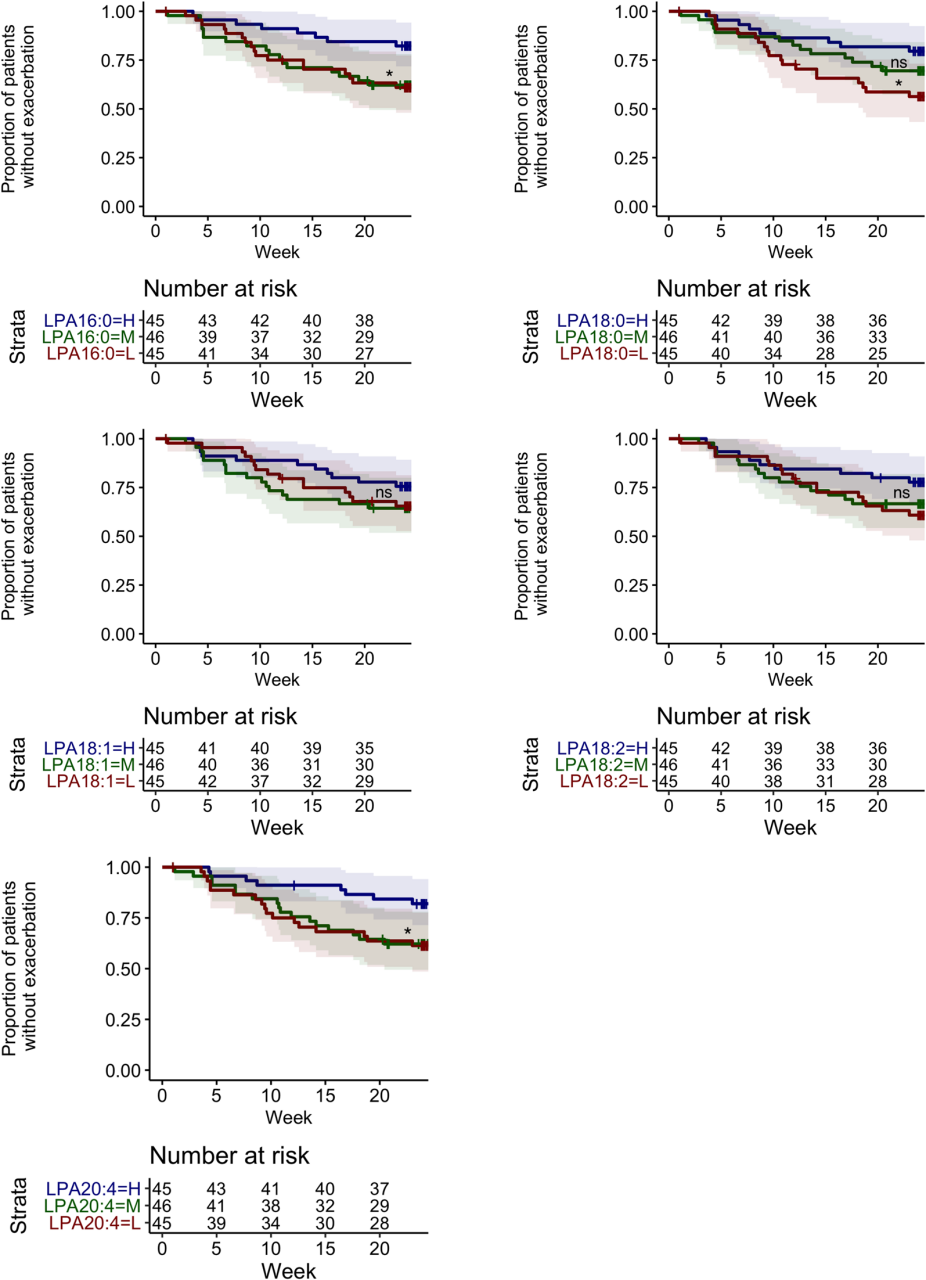
Time to first exacerbation by baseline LPA profile. Baseline LPA profile was fitted to a Cox proportional hazards regression model adjusted for the following covariates: number of exacerbations within the last 12 months, smoking status, geographical region, baseline COPD medications, and sex. L = lowest-; M = medium-; H = highest-tertile of the respective LPA levels. *P*-values compared L or M to H subgroup. *p < 0.05; ns, not significant

Time to first exacerbation was significantly earlier in patients with low LPA16:0, LPA18:0, or LPA20:4; or medium LPA16:0 or LPA20:4, as compared to the respective LPA-high subgroups: (hazard ratio (95% CI)) LPA16:0-low = 2.6 (1.1–6.0) (*p* = 0.028), LPA16:0-medium = 2.7 (1.2–6.3) (*p* = 0.020); LPA18.0-low = 2.5 (1.1–5.4) (*p* = 0.027); LPA20.4-low = 2.8 (1.2–6.6) (*p* = 0.017), and LPA20:4-medium = 2.7 (1.2–6.4) (*p* = 0.021) (Fig. 3).

**Fig. 4:**
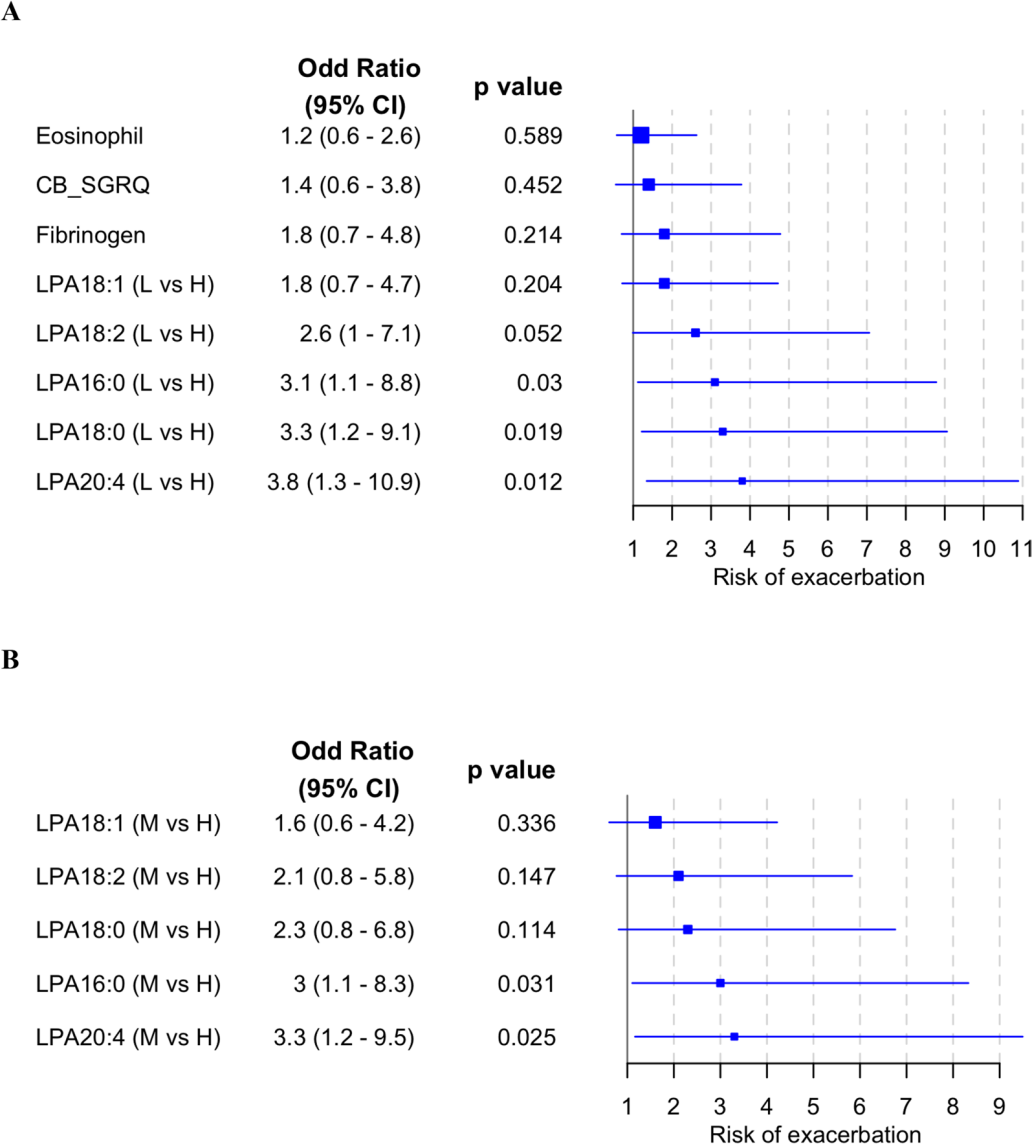
Risk of exacerbation by baseline LPA profile. Baseline LPA profile was fitted to a multivariate logistic regression model adjusted for the following covariates: number of exacerbations within the last 12 months, smoking status, geographical region, baseline COPD medications, and sex. An odd ratio above 1 denotes higher odds of exacerbation (**a**) in patients with the lowest tertile LPA compared to highest tertile LPA (L vs H); blood eosinophils ≥ 200 cells/µL compared to < 200 cells/µL; fibrinogen ≥ 3.5 g/L compared to < 3.5 g/L; or with chronic bronchitis compared to no chronic bronchitis; (**b**) in patients with the medium tertile LPA compared to highest tertile LPA (M vs H). CB_SGRQ, chronic bronchitis—St. George’s Respiratory Questionnaire COPD

Patients with low LPA16:0, LPA18:0, or LPA20:4; or medium LPA16:0 or LPA20:4, had significantly higher odds of having an exacerbation within the 24-week follow-up period (odd ratio (95% CI)): LPA16:0-low = 3.1 (1.1–8.8) (*p* = 0.03); LPA16:0-medium = 3.0 (1.1–8.3) (*p* = 0.031); LPA18:0-low = 3.3 (1.2–9.1) (*p* = 0.019); LPA20:4-low = 3.8 (1.3–10.9) (*p* = 0.012) LPA20:4-medium = 3.3 (1.2–9.5) (*p* = 0.025), as compared to the respective LPA-high patients (Fig. 4). As blood eosinophils above 300 cells/µL [8], fibrinogen above 3.5 g/L [13], and the presence of chronic bronchitis have been reported to be associated with exacerbation, they were included as benchmarks. Since very few patients had eosinophils at or above 300 cells/µL (n = 27), 200 cells/µL was used as the cutoff to subgroup patients. There was no significant increase in exacerbation risk in patients with high blood eosinophils (≥ 200 cells/µL) (n = 53), high fibrinogen (≥ 3.5 g/L) (n = 55) or with chronic bronchitis (n = 107), compared to patients with low blood eosinophils (< 200 cells/µL), low fibrinogen (< 3.5 g/L), or without chronic bronchitis, respectively (Fig. 4).

A lower proportion of exacerbations in LPA20:4-high patients were treated with systemic corticosteroids (76.5%), as compared to 90.6% and 100% in the LPA20:4-medium and -high patients, respectively (*p* = 0.0087) (Table 3). There was no significant difference in the proportion of hospitalized or antibiotic-treated exacerbations among the LPA subgroups (Table 3). Exacerbation duration was not significantly different among the LPA subgroups (Additional file 1: Figure S2). Samples were not collected during exacerbation to examine the changes in LPA levels surrounding an exacerbation, but time since last exacerbation before the patients enrolled into the study was recorded. Baseline LPA levels were not associated with time since last exacerbation in univariate or multivariate regression adjusting for the aforementioned covariates (Additional file 1: Figure S3).

**Table 3:** Exacerbation severity and treatment by baseline biomarker profile

	Number of exacerbation	Hospitalized exacerbation N (%)	*p*-value	Exacerbation treated with antibiotics N (%)	*p*-value	Exacerbation treated with systemic corticosteroids N (%)	*p*-value
LPA16:0							
Low	32	6 (18.8)		28 (87.5)		29 (90.6)	
Medium	31	4 (12.9)	0.42	23 (74.2)	0.058	29 (93.5)	0.89
High	19	1 (5.3)		11 (57.9)		17 (89.5)	
LPA18:0							
Low	31	4 (12.9)		24 (77.4)		28 (90.3)	1.0
Medium	30	5 (16.7)	0.85	24 (80.0)	0.58	28 (93.3)	
High	21	2 (9.5)		14 (66.7)		19 (90.5)	
LPA18:1							
Low	25	2 (8.0)		22 (88.0)		22 (88.0)	
Medium	34	7 (20.6)	0.36	26 (76.5)	0.10	33 (97.1)	0.32
High	23	2 (8.7)		14 (60.9)		20 (87.0)	
LPA18:2							
Low	29	4 (13.8)		21 (72.4)		27 (93.1)	
Medium	30	4 (13.3)	1.0	26 (86.7)	0.18	27 (90.0)	1.0
High	23	3 (13.0)		15 (65.2)		21 (91.3)	
LPA20:4							
Low	32	5 (15.6)		25 (78.1)		29 (90.6)	
Medium	33	4 (12.1)	0.92	24 (72.7)	0.944	33 (100)	0.0087*
High	17	2 (11.8)		13 (76.5)		13 (76.5)	

Fisher’s Exact test *p*-values shown

### LPA and lipidomics

To investigate the potential metabolic shifts underlying increased exacerbation rate and risk in LPA subgroups, lipidomics were compared between LPA low versus high patients since there were consistent differences in exacerbation metrics between these two extreme subgroups. LPE and LPC, the precursors of LPA, were lower in LPA-low compared to the high subgroups (*p* < 0.05, FDR < 0.05) (Additional file 1: Figure S4). A total of 507 lipid species were detectable in at least 90% of the patients; comparison of these lipid species between LPA-low and high subgroups showed only modest changes, where a number of lipid species showed differential expression before adjusting for multiplicity (*p* < 0.05), but none of these lipid species were consistently decreased or increased across all LPA species (Additional file 1: Figure S4).

## Discussion

Effective identification of high-risk patients prone to exacerbation is critical for the management of the disease as well as the acceleration of drug development. In this study, we identified serum LPA species as prognostic biomarkers of exacerbation. Patients with low and medium levels of LPA (LPA16:0, 20:4) had significantly higher rate and risk of exacerbation; and earlier time to first exacerbation, compared to the respective LPA-high patients. A higher proportion of exacerbations in LPA20:4-low and -medium patients were treated with systemic corticosteroids compared to the LPA20:4-high patients. We observed similar trends for the other LPA species (LPA18:0, 18:1, 18:2), where patients with low and medium levels of LPA18:0 and 18:2 had higher exacerbation rate; but only LPA18:0-low patients had a significant increase in exacerbation risk and earlier time to first exacerbation compared to the LPA18:0-high subgroup. LPA levels were not significantly associated with other baseline clinical variables in this study.

Previously we reported that the majority of the LPA species, except LPA20:4, are higher in a small cohort of COPD patients compared to healthy controls [26]. It is unclear if COPD patients with low levels of LPA16 and LPA18 species in this study have significantly higher levels of these LPAs relative to healthy individuals, as samples from age- and sex-matched healthy subjects were not tested together with the COPD samples in the current study. However, it is plausible that low levels of LPAs in COPD subjects might be linked to infection or unresolved inflammation. Decrease in systemic levels of lipids including LPA have been reported in COPD and patients with lung infection. Plasma LPC, precursor of LPA, was significantly lower in patients with COPD and the reduction was more pronounced in patients with pneumonia compared to healthy control [28], in line with the observation that LPC in cell lines were markedly decreased post *S. aureus*-infection [28]. Similar results were replicated in independent studies, where blood LPC or LPA levels were significantly reduced in patients with H7N9 infection [29] and sepsis [30]; importantly, decreased LPC/PC ratio predicted sepsis-related mortality [30]. Unsurprisingly, systemic LPC was decreased in a murine model of peritoneal sepsis caused by *A. baumannii*, and the LPC depletion coincided with inflammatory cytokine release [31]. These results underscore the importance of the LPC-LPA axis in infection, where lower levels of these lipids were observed following infection and associated with the worse outcomes and inflammatory cytokines. LPA modulates immune responses by directly impacting various functional aspects of neutrophils, monocytes, macrophages, dendritic cells, as well as T cells [32]. Neutrophils from pneumonia patients had increased chemotaxis to LPA due to the increased expression of LPA receptor 1 [33]. LPA promotes monocyte migration [34] and mediates the differentiation of monocytes to macrophages [35], where reactive oxygen species are released from macrophages to eliminate pathogens [36]. Additionally, monocyte recruitment via LPA signaling has been shown to be crucial for the resolution of tissue inflammation [37]. Extrapolating these observations to this study, our data suggests that the increase in exacerbation and earlier time to first exacerbation in COPD patients with lower levels of LPA in this study could be related to microbial colonization, reduced neutrophil chemotaxis and monocyte differentiation into macrophages to effectively eliminate pathogens, or impairment in resolving tissue inflammation, all of which could lead to prolonged inflammation. Interestingly, we observed that a higher proportion of exacerbations in patients with lower levels of LPA20:4 were treated with systemic corticosteroids. Unfortunately, samples were not collected during exacerbation in the study to characterize the exacerbation triggers.

In vitro, ATX binds to exosomes to generate LPA16:0, 18:0, and 18:1, which were carried in the vesicles; LPA18:2 and 20:4 were also generated but to a lesser degree [38]. It is thought that ATX-LPA bound to exosomes were retained within the vesicles till they reached the target cells to mediate signaling [38]. Exosomes are stable in systemic circulation due to the lipid bilayer and it has been reported that specific exosome levels are elevated during COPD exacerbation [39] and associated with systemic inflammation [40]. It is possible that patients with low levels of detectable or “free” LPA, had the LPA sequestered in exosomes to be transported to the site of injury in the lung to exert biological functions locally, and the increased exacerbation observed in patients with low levels of LPA could be a consequence of the underlying lung injury.

LPA mediates its downstream effects through receptor activation. LPA receptors are expressed at different levels on different cell types [21]. The intensity of receptor activation by the LPA species coupled with the type of LPA receptors activated are likely to dictate the net outcome. In line with this, literature has reported both the protective as well as pathogenic roles of LPA in the airway. LPA receptors are required to maintain epithelial barrier function, control allergic lung inflammation [41, 42], and support alveolarization [43]. Related to lung pathology, activation of lung LPA receptors leads to fibrosis [23, 44], epithelial cell apoptosis [45], and promotes inflammatory cytokine production and neutrophilic infiltration [46]. LPA can also bind to non-G-protein-coupled receptors such as RAGE and PPARγ to mediate vascular inflammation and platelet activation [47]. It is unclear if the differences in the prognostic properties among the LPA species observed in this study were due to the differential binding affinity of the LPA species to the different receptor subtypes. Further work is needed to investigate the molecular signaling pathways elicited by the individual LPA species, to understand why LPA16:0 and 20:4 were prognostic across the various metrics of exacerbation, but not the other LPA species.

The exacerbation status of the patients varies from year to year and there are very few patients who consistently have exacerbations every year [48]. Our data suggests that LPA-high patients might belong to a varying category as these patients had low on-study exacerbation rate despite the fact that all of them had at least one exacerbation in the prior year. In contrast, patients with lower LPA levels, especially LPA16:0 and 20:4, had significant odds of having an exacerbation. It is unclear if the underlying pathobiology of these high-risk patients is different from those without yearly exacerbation, our data suggested that LPA could have an important role in driving exacerbation in patients who had exacerbations in consecutive years. Although exacerbation metrics were different between LPA-low and high subgroups, lipidomic profiling did not show consistent changes in lipid classes or lipid species between the subgroups, suggesting that the LPA species could be the more sensitive markers to identify these high-risk patients. Moreover, LPA is not correlated with known inflammation biomarker fibrinogen, indicating that these LPA levels do not reflect systemic inflammation but uniquely dysregulated LPA pathway.

Since fibrinogen is a non-specific marker of systemic inflammation, it was not significantly associated with exacerbation risk in this study; a finding in line with a previous report of two large COPD cohorts [7]. Our data did not show a positive association between LPA and FEV_1_%predicted as reported by Naz et al. [25], potentially due to the differences in detection assays or other patient-specific factors as the number of patients included here was larger. The study has several limitations. First, this was a post-hoc analysis of a clinical trial and sampling was incomplete. Nevertheless, the demographics and exacerbation events captured were representative of the overall placebo population. Second, the differences in exacerbation outcomes were based on the sex-specific tertile level of the LPA used to subgroup patients. Additional work is needed to assess the optimal cutoff. Finally, although there is mounting evidence to implicate the LPA pathway in the pathogenesis of inflammatory lung diseases, the relationship between systemic LPA levels and the lung compartment has not been examined as lung sampling was not collected in this study. Neither were samples collected during exacerbation to characterize exacerbation etiology. Measurements of autotaxin and other proteins involved in LPA metabolism were also unavailable to elucidate potential mechanisms.

## Conclusion

We developed a mass spectrometry method to quantify the main serum LPA species in COPD patients from a well characterized clinical study. LPA levels were not associated with baseline clinical metrics or demographics except for sex. These LPA biomarkers were prognostic as patients with lower levels of specific LPA species had increased rate and risk of exacerbation and earlier time to first exacerbation compared to the LPA-high patients, potentially due to a multitude of effects of LPA on immune regulation which could impair pathogen clearance or the resolution of inflammation. These LPA biomarkers could potentially be used to identify high-risk patients who have exacerbations in consecutive years. Further work is needed to understand the mechanistic connection between LPA and COPD exacerbation.

## Supplementary Information

**Additional file 1**. **Table S1.** Patient baseline characteristics by LPA18:0 subgroups. **Table S2.** Patient baseline characteristics by LPA18:1 subgroups. **Table S3.** Patient baseline characteristics by LPA18:2 subgroups. **Table S4.** Patient baseline characteristics by LPA20:4 subgroups. **Table S5.** List of investigator and Institutional Review Board / Ethics Committee approval. **Figure S1.** Baseline serum LPA levels by (**A**) sex, (**B**) smoking status, and (**C**) COPD medications; double = ICS with one bronchodilator; triple = ICS with two bronchodilators. Median and interquartile range shown as boxplot. M, male; F, female. **p < 0.005; *** p < 0.001; ns, not significant, Student t-test on log_2_ transformed LPA. **Figure S2.** Differences in exacerbation duration among LPA subgroups. Median and interquartile range shown as boxplot. L = lowest-; M = medium-; H = highest-tertile of the respective LPA levels; N, number of exacerbation events. Kruskal-Wallis p-values shown. **Figure S3.** Baseline serum LPA levels and time since last exacerbation before study entry. P-values showed the association of LPA with time since last exacerbation before patients were enrolled into the study in a multivariate regression adjusted for the following covariates: number of exacerbations within the last 12 months, smoking status, geographical region, baseline COPD medications, and sex. P = ns, not significant; uM, microMolar. **Figure S4.** Differential lipid expression by baseline biomarker profile. (**A**) Profiling of 12 classes of lipids in LPA low versus high patients. The *x* axis denotes the average log_2_ (analyte abundance in low/analyte abundance in high); values less than 0 indicate a decrease, and values greater than 0 an increase, in low versus high patients. Green bars denote unadjusted p-value < 0.05; orange bars denote false discovery rate < 0.05. CE, cholesteryl esters; CER, ceramides; DAG, diacylglycerols; DCER, dihydroceramides; HCER, hexosylceramides; LCER, lactosylceramides; LPC, lysophosphatidylcholines; LPE, lysophosphatidylethanolamines; PC, phosphatidylcholines; PE, phosphatidylethanolamines; SM, sphingomyelins; TAG, triacylglycerols. (**B**) Volcano plots showing lipid species in LPA low versus high patients. The x axis denotes log2(analyte abundance in low/analyte abundance in high), and the y axis indicates the -log10(unadjusted p-value). Colored circles denote unadjusted p-value<0.05; red circles denote lipid species with higher abundance in LPA-low compared to LPA-high (fold change > 0.5); blue circles denote lipid species with lower abundance in LPA-low compared to LPA-high (fold change > 0.5).

## Data Availability

The datasets used and/or analyzed during the current study are available from the corresponding author on reasonable request.

## Abbreviations

ATX: Autotaxin
CB-SGRQ: Chronic bronchitis derived from St George’s Respiratory Questionnaire-COPD
COPD: Chronic obstructive pulmonary disease
CE: Cholesteryl esters
CER: Ceramides
DAG: Diacylglycerols
DCER: Dihydroceramides
GOLD: Global Initiative for Chronic Obstructive Lung Disease
HCER: Hexosylceramides
LC–MS/MS: Liquid chromatography-mass spectrometry/ mass spectrometry
LCER: Lactosylceramides
LPA: Lysophosphatidic acid
LPC: Lysophosphatidylcholines
LPE: Lysophosphatidylethanolamines
PC: Phosphatidylcholines
PE: Phosphatidylethanolamines
SGRQ-C: St George’s Respiratory Questionnaire—COPD
SM: Sphingomyelins
TAG: Triacylglycerols
