# Supplementary material for: Lysophosphatidic acid species are associated with exacerbation in chronic obstructive pulmonary disease
Source: BMC Pulm Med. 2021 Sep 23;21:301. doi: 10.1186/s12890-021-01670-9 (PMC8461999; doi:10.1186/s12890-021-01670-9)
Supplement: Supplementary file 1 — Additional file 1. Table S1. Patient baseline characteristics by LPA18:0 subgroups. Table S2. Patient baseline characteristics by LPA18:1 subgroups. Table S3. Patient baseline characteristics by LPA18:2 subgroups. Table S4. Patient baseline characteristics by LPA20:4 subgroups. Table S5. List of investigator and Institutional Review Board / Ethics Committee approval. Figure S1. Baseline serum LPA levels by (A) sex, (B) smoking status, and (C) COPD medications; double = ICS with one bronchodilator; triple = ICS with two bronchodilators. Median and interquartile range shown as boxplot. M, male; F, female. **p < 0.005; *** p < 0.001; ns, not significant, Student t-test on log2 transformed LPA. Figure S2. Differences in exacerbation duration among LPA subgroups. Median and interquartile range shown as boxplot. L = lowest-; M = medium-; H = highest-tertile of the respective LPA levels; N, number of exacerbation events. Kruskal-Wallis p-values shown. Figure S3. Baseline serum LPA levels and time since last exacerbation before study entry. P-values showed the association of LPA with time since last exacerbation before patients were enrolled into the study in a multivariate regression adjusted for the following covariates: number of exacerbations within the last 12 months, smoking status, geographical region, baseline COPD medications, and sex. P = ns, not significant; uM, microMolar. Figure S4. Differential lipid expression by baseline biomarker profile. (A) Profiling of 12 classes of lipids in LPA low versus high patients. The x axis denotes the average log2 (analyte abundance in low/analyte abundance in high); values less than 0 indicate a decrease, and values greater than 0 an increase, in low versus high patients. Green bars denote unadjusted p-value < 0.05; orange bars denote false discovery rate < 0.05. CE, cholesteryl esters; CER, ceramides; DAG, diacylglycerols; DCER, dihydroceramides; HCER, hexosylceramides; LCER, lactosylceramides; LPC, lysophosphatidylcholines; [file 12890_2021_1670_MOESM1_ESM.pdf]

Lysophosphatidic acid species are associated with exacerbation in chronic obstructive pulmonary disease

Supplementary Information

Table S1. Patient baseline characteristics by LPA18:0 subgroups

LPA18:0<br><br>LPA18:0 tertile concentration (µM)<br>Male<br>Female	Low<br>N=45<br><br><br><0.021<br><0.029	Medium<br>N=46<br><br><br>0.021 to <0.03<br>0.029 to <0.046	High<br>N=45<br><br><br>≥0.030<br>≥0.046	p-value
Age (years)	62.7 (8.3)	65.2 (6.8)	65.7 (6.5)	0.10
Body mass index	27.8 (5.1)	26.2 (5)	27.4 (5.2)	0.27
Former smokers, %	28 (62)	24 (52)	24 (53)	0.60
Post-bronchodilator FEV_1<br>Absolute (L)<br>% predicted	 1.3 (0.5)<br>44.8 (16.9)	 1.3 (0.4)<br>47.5 (14.5)	 1.3 (0.4)<br>47.6 (14.4)	 0.94<br>0.63
Post-bronchodilator FEV_1/FVC ratio	0.5 (0.1)	0.5 (0.1)	0.5 (0.1)	0.74
SGRQ-C score	58.5 (16.3)	58.4 (17.5)	55.8 (19.4)	0.72
GOLD, %<br>Stage II<br>Stage III<br>Stage IV	 18 (40)<br>15 (33.3)<br>12 (26.7)	 24 (52.2)<br>15 (32.6)<br>7 (15.2)	 20 (44.4)<br>19 (42.2)<br>6 (13.3)	 <br>0.43
Patients with chronic bronchitis, %	35 (78)	38 (83)	34 (76)	0.72
Patients with severe exacerbation in previous 12 months, %	15 (33)	11 (24)	11 (24)	0.53
Median blood eosinophil count (cells/µL)	180 (130)	175 (120)	180 (215)	0.64
Median fibrinogen (g/L)	3.6 (1.5)	3.7 (1.2)	3.5 (0.9)	0.36

Data are n (%), mean (SD), or median (IQR). FEV_1, forced expiratory volume in 1 second; FVC, forced vital capacity; SGRQ-C, St. George's Respiratory Questionnaire COPD.

Table S2. Patient baseline characteristics by LPA18:1 subgroups

LPA18:1<br><br>LPA18:1 tertile concentration (μM)<br>Male<br>Female	Low<br>N=45<br><br><br><0.11<br><0.14	Medium<br>N=46<br><br><br>0.11 to <0.17<br>0.14 to <0.25	High<br>N=45<br><br><br>≥0.17<br>≥0.25	p-value
Age (years)	62.6 (7.4)	65.7 (6.9)	65.4 (7.5)	0.080
Body mass index	27.8 (5)	25.7 (5.1)	28 (5.2)	0.065
Former smokers, %	23 (51)	30 (65)	23 (51)	0.30
Post-bronchodilator FEV_1<br>Absolute (L)<br>% predicted	 1.3 (0.5)<br>47.2 (17)	 1.3 (0.5)<br>44.9 (15.3)	 1.3 (0.4)<br>47.8 (13.3)	 0.81<br>0.63
Post-bronchodilator FEV_1/FVC ratio	0.5 (0.1)	0.5 (0.1)	0.5 (0.1)	0.77
SGRQ-C score	57 (15.5)	56.4 (18.4)	59.2 (19.2)	0.74
GOLD, %<br>Stage II<br>Stage III<br>Stage IV	 22 (48.9)<br>14 (31.1)<br>9 (20)	 17 (37)<br>19 (41.3)<br>10 (21.7)	 23 (51.1)<br>16 (35.6)<br>6 (13.3)	 <br>0.60
Patients with chronic bronchitis, %	36 (80)	36 (78)	35 (78)	0.90
Patients with severe exacerbation in previous 12 months, %	13 (29)	12 (26)	12 (27)	0.97
Median blood eosinophil count (cells/μL)	170 (105)	190 (130)	180 (255)	0.44
Median fibrinogen (g/L)	3.5 (1.1)	3.5 (1.3)	3.5 (1)	0.96

Data are n (%), mean (SD), or median (IQR). FEV_1, forced expiratory volume in 1 second; FVC, forced vital capacity; SGRQ-C, St. George's Respiratory Questionnaire COPD.

Table S3. Patient baseline characteristics by LPA18:2 subgroups

LPA18:2<br><br>LPA18:2 tertile concentration (µM)<br>Male<br>Female	Low<br>N=45<br><br><br><br><0.42<br><0.56	Medium<br>N=46<br><br><br><br>0.42 to <0.66<br>0.56 to <0.89	High<br>N=45<br><br><br><br>≥0.66<br>≥0.89	p-value
Age (years)	63.2 (7.6)	64.5 (7.7)	66 (6.6)	0.20
Body mass index	28.1 (5.1)	26.1 (4.8)	27.2 (5.4)	0.19
Former smokers, %	30 (67)	22 (48)	24 (53)	0.19
Post-bronchodilator FEV_1<br>Absolute (L)<br>% predicted	 1.3 (0.5)<br>45.3 (16.6)	 1.3 (0.4)<br>46.1 (15.2)	 1.4 (0.4)<br>48.5 (14)	 0.67<br>0.59
Post-bronchodilator FEV_1/FVC ratio	0.5 (0.1)	0.5 (0.1)	0.5 (0.1)	0.40
SGRQ-C score	57.4 (15.6)	59.3 (17.7)	55.9 (19.7)	0.67
GOLD, %<br>Stage II<br>Stage III<br>Stage IV	 19 (42.2)<br>15 (33.3)<br>11 (24.4)	 21 (45.7)<br>16 (34.8)<br>9 (19.6)	 22 (48.9)<br>18 (40)<br>5 (11.1)	 <br>0.60
Patients with chronic bronchitis, %	36 (80)	38 (83)	33 (73)	0.52
Patients with severe exacerbation in previous 12 months, %	17 (38)	10 (22)	10 (22)	0.17
Median blood eosinophil count (cells/µL)	160 (122.5)	190 (130)	175 (197.5)	0.62
Median fibrinogen (g/L)	3.8 (1.7)	3.6 (1)	3.5 (0.6)	0.42

Data are n (%), mean (SD), or median (IQR). FEV_1, forced expiratory volume in 1 second; FVC, forced vital capacity; SGRQ-C, St. George's Respiratory Questionnaire COPD.

Table S4. Patient baseline characteristics by LPA20:4 subgroups

LPA20:4<br><br>LPA20:4 tertile concentration (µM)<br>Male<br>Female	Low<br>N=45<br><br><br><6.8<br><6.7	Medium<br>N=46<br><br><br>6.8 to <9.5<br>6.7 to <11.8	High<br>N=45<br><br><br>≥9.5<br>≥11.8	p-value
Age (years)	64.4 (7.4)	64.5 (6.8)	64.7 (7.8)	0.98
Body mass index	27.2 (4.8)	26.5 (5.7)	27.7 (4.9)	0.58
Former smokers, %	31 (69)	22 (48)	22 (49)	0.10
Post-bronchodilator FEV_1				
Absolute (L)	1.3 (0.5)	1.3 (0.5)	1.3 (0.5)	0.59
% predicted	44.4 (14.9)	46.3 (16.2)	49.2 (14.5)	0.33
Post-bronchodilator FEV_1/FVC ratio	0.5 (0.1)	0.5 (0.1)	0.5 (0.1)	0.44
SGRQ-C score	57.2 (18.9)	58 (17)	57.3 (17.6)	0.97
GOLD, %				
Stage II	18 (40)	21 (45.7)	23 (51.1)	
Stage III	17 (37.8)	14 (30.4)	18 (40)	0.31
Stage IV	10 (22.2)	11 (23.9)	4 (8.9)	
Patients with chronic bronchitis, %	31 (69)	40 (87)	36 (80)	0.18
Patients with severe exacerbation in previous 12 months, %	17 (38)	10 (22)	10 (22)	0.17
Median blood eosinophil count (cells/µL)	190 (150)	170 (110)	180 (145)	0.93
Median fibrinogen (g/L)	3.5 (1.2)	3.6 (1.7)	3.6 (1)	0.73

Data are n (%), mean (SD), or median (IQR). FEV_1, forced expiratory volume in 1 second; FVC, forced vital capacity; SGRQ-C, St. George's Respiratory Questionnaire COPD.

Table S5. List of investigator and Institutional Review Board / Ethics Committee approval

Site #	Investigator	IRB/EC name and address (if available)	Approval date
285134	N. Hanania	Baylor College of Medicine IRB	13-Oct-2015
285239	M. Seep	Quorum Review IRB	30-Oct-2015
285382	Z. Cseke	Medical Research Council, Ethics Committee for Clinical Pharmacology, Arany J. u. 6-8., 1051, Budapest, HUNGARY	12-Oct-2015
285384	Z. Szalai	Medical Research Council, Ethics Committee for Clinical Pharmacology, Arany J. u. 6-8., 1051, Budapest, HUNGARY	12-Oct-2015
285387	W. Pierzchala	Komisja Bioetyczna przy Slaskiej Izbie Lekarskiej w Katowicach, 49a, Grazynskiego, 40-126, Katowice, Poland	26-Oct-2015
285389	M. Póczi	Medical Research Council, Ethics Committee for Clinical Pharmacology, Arany J. u. 6-8., 1051, Budapest, HUNGARY	12-Oct-2015
285390	M. Taseva	Ethics Committee for Multicenter Trials, 5, Sveta Nedelia Sq., 1000, Sofia, BULGARIA	11-Nov-2015
285391	S. Sotirov	Ethics Committee for Multicenter Trials, 5, Sveta Nedelia Sq., 1000, Sofia, BULGARIA	11-Nov-2015
285393	H. Metev	Ethics Committee for Multicenter Trials, 5, Sveta Nedelia Sq., 1000, Sofia, BULGARIA	11-Nov-2015
285394	D. Dimov	Ethics Committee for Multicenter Trials, 5, Sveta Nedelia Sq., 1000, Sofia, BULGARIA	11-Nov-2015
285395	K. Aleksandrov	Ethics Committee for Multicenter Trials, 5, Sveta Nedelia Sq., 1000, Sofia, BULGARIA	11-Nov-2015
285397	L. Molhar	Medical Research Council, Ethics Committee for Clinical Pharmacology, Arany J. u. 6-8., 1051, Budapest, HUNGARY	15-Oct-2015

285477	A. Antczak	Komisja Bioetyczna przy Slaskiej Izbie Lekarskiej w Katowicach, 49a, Grazynskiego, 40-126, Katowice, Poland	16-Nov-2015
285553	P. Ilieva	Ethics Committee for Multicenter Trials, 5, Sevta Nedelia Sq., 1000, Sofia, BULGARIA	11-Nov-2015
285556	É. Radeczk	Medical Research Council, Ethics Committee for Clinical Pharmacology, Arany J. u. 6-8., 1051, Budapest, HUNGARY	12-Oct-2015
285995	M. De Salvo	Comité Independiente de Ética para Ensayos en Farmacología Clínica del Centra Médico Dra. De Salvo, Avenida Cabildo 1536 5°B, C1426ABP, Ciudad Autónoma de Buenos Aires, ARGENTINA	18-Jul-2015
285996	L. Wehbe	Comité de Ética en Inventigación Instituto Ave Pulmo, Carlos Alvear 3345, 7600, Mar del Plata, ARGENTINA	06-Oct-2015
285997	M. Mailo	Comité Independiente de Ética para Ensayos en Farmacología Clínica del Centra Médico Dra. De Salvo, Avenida Cabildo 1536 5°B, C1426ABP, Ciudad Autónoma de Buenos Aires, ARGENTINA	29-Oct-2015
287000	A. Ramírez Venegas	Comite de Etica en Investigacion de Mexico Centre for Clinical Research SA de CV, Amores No. 709, Col. Del Valle, 03100, Distrito Federal, MEXICO	29-Sep-2015
287004	J. Milanowski	Komisja Bioetyczna przy Slaskiej Izbie Lekarskiej w Katowicach, 49a, Grazynskiego, 40-126, Katowice, Poland	16-Nov-2015
287043	J. Schlezák	Medical Research Council, Ethics Committee for Clinical Pharmacology, Arany J. u. 6-8., 1051, Budapest, HUNGARY	12-Oct-2015
287046	E. Rybicka-Liszewska	Komisja Bioetyczna przy Slaskiej Izbie Lekarskiej w Katowicach, 49a, Grazynskiego, 40-126, Katowice, Poland	26-Oct-2015
287441	C.S. Ulrick	Videnskabsetiske Komiteer Region Hovedstaden, Kongens Vaenge 2, 3400, Hilleroed, DENMARK	08-Jan-2016

287515	A. Colli Dominguez	OSMO, S.C.; Comité de ética en investigación y comité de investigación, Himboldt 302, col. centro, C.P. 68000, Oaxaca, OAXACA, MEXICO	28-Sep-2015
291889	R. Dobek	Komisja Bioetyczna przy Slaskiej Izbie Lekarskiej w Katowicach, 49a, Grazynskiego, 40-126, Katowice, Poland	15-Mar-2016
291890	I. Albert	Medical Research Council, Ethics Committee for Clinical Pharmacology, Arany J. u. 6-8., 1051, Budapest, HUNGARY	08-Apr-2016
292047	M. Papp	Medical Research Council, Ethics Committee for Clinical Pharmacology, Arany J. u. 6-8., 1051, Budapest, HUNGARY	08-Apr-2016

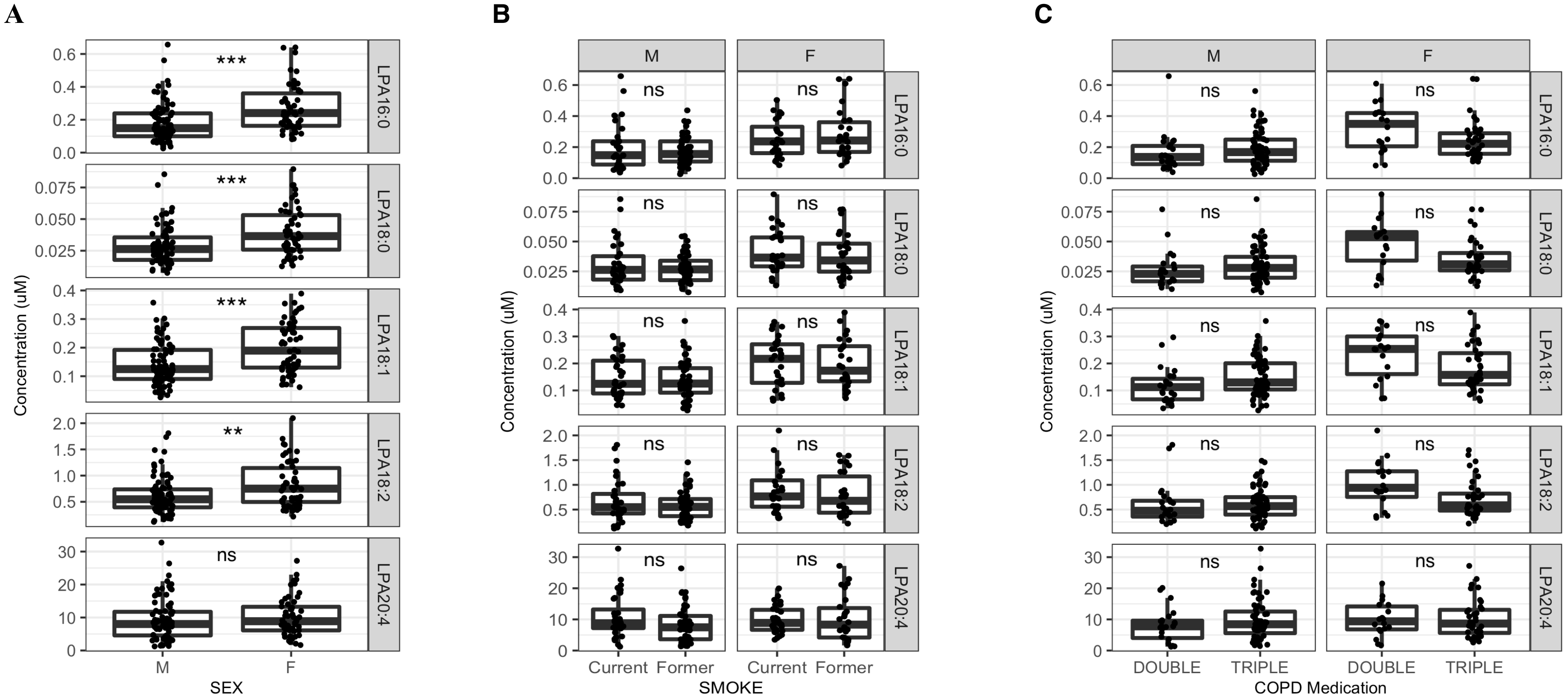

Figure S1. Baseline serum LPA levels by (A) sex, (B) smoking status, and (C) COPD medications; double=ICS with one bronchodilator; triple=ICS with two bronchodilators. Median and interquartile range shown as boxplot. M, male; F, female. **p<0.005; ***p<0.001; ns, not significant, Student t-test on log2 transformed LPA.

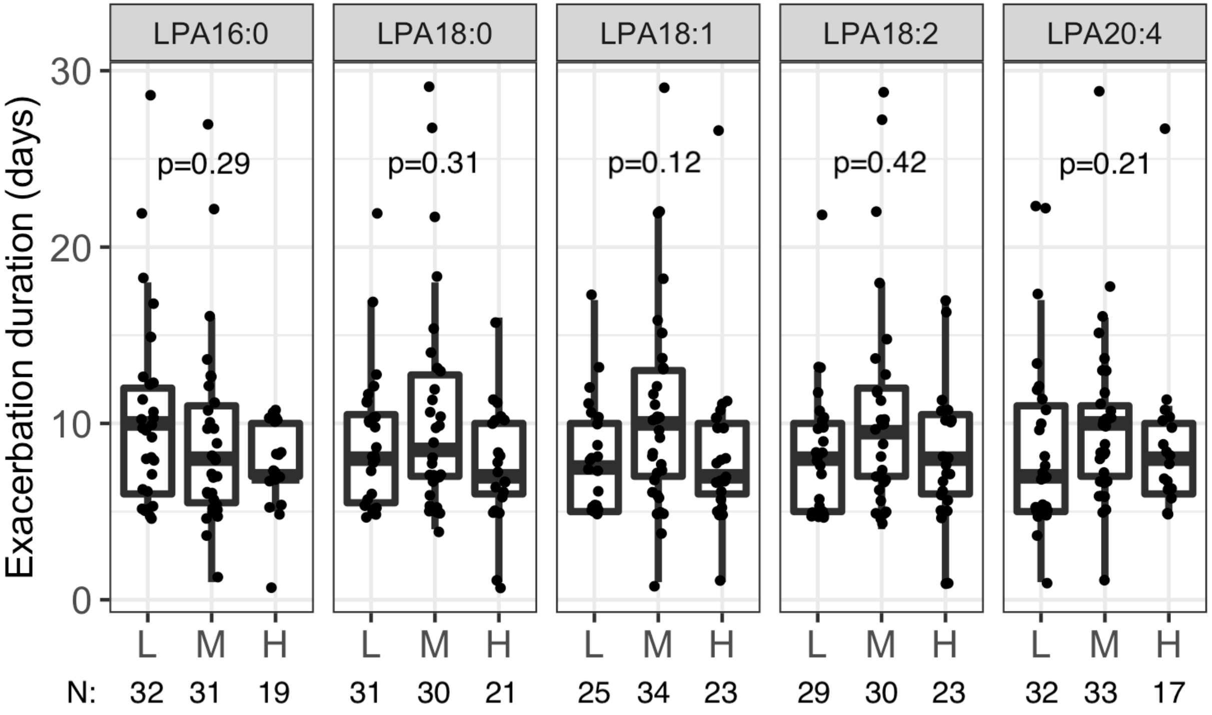

Figure S2. Differences in exacerbation duration among LPA subgroups. Median and interquartile range shown as boxplot. L=lowest-; M=medium-; H=highest-tertile of the respective LPA levels; N, number of exacerbation events. Kruskal-Wallis p-values shown.

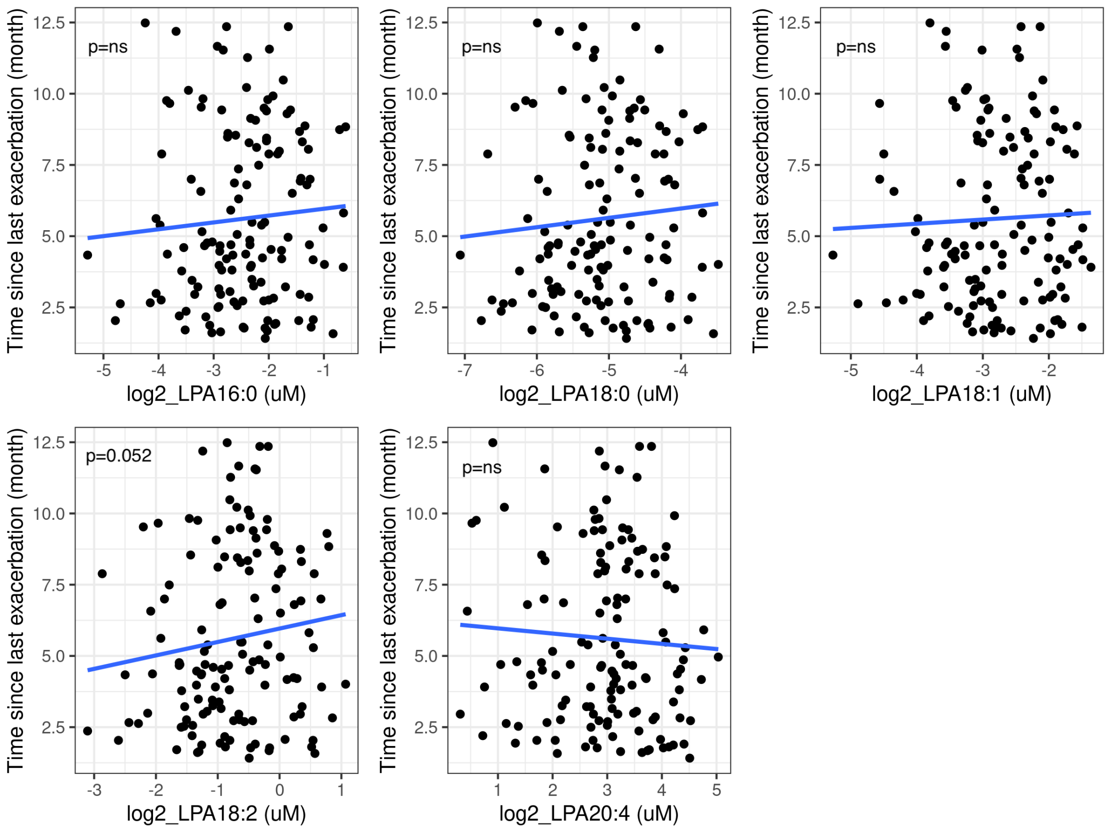

Figure S3. Baseline serum LPA levels and time since last exacerbation before study entry. P-values showed the association of LPA with time since last exacerbation before patients were enrolled into the study in a multivariate regression adjusted for the following covariates: number of exacerbations within the last 12 months, smoking status, geographical region, baseline COPD medications, and sex. P=ns, not significant; uM, microMolar.

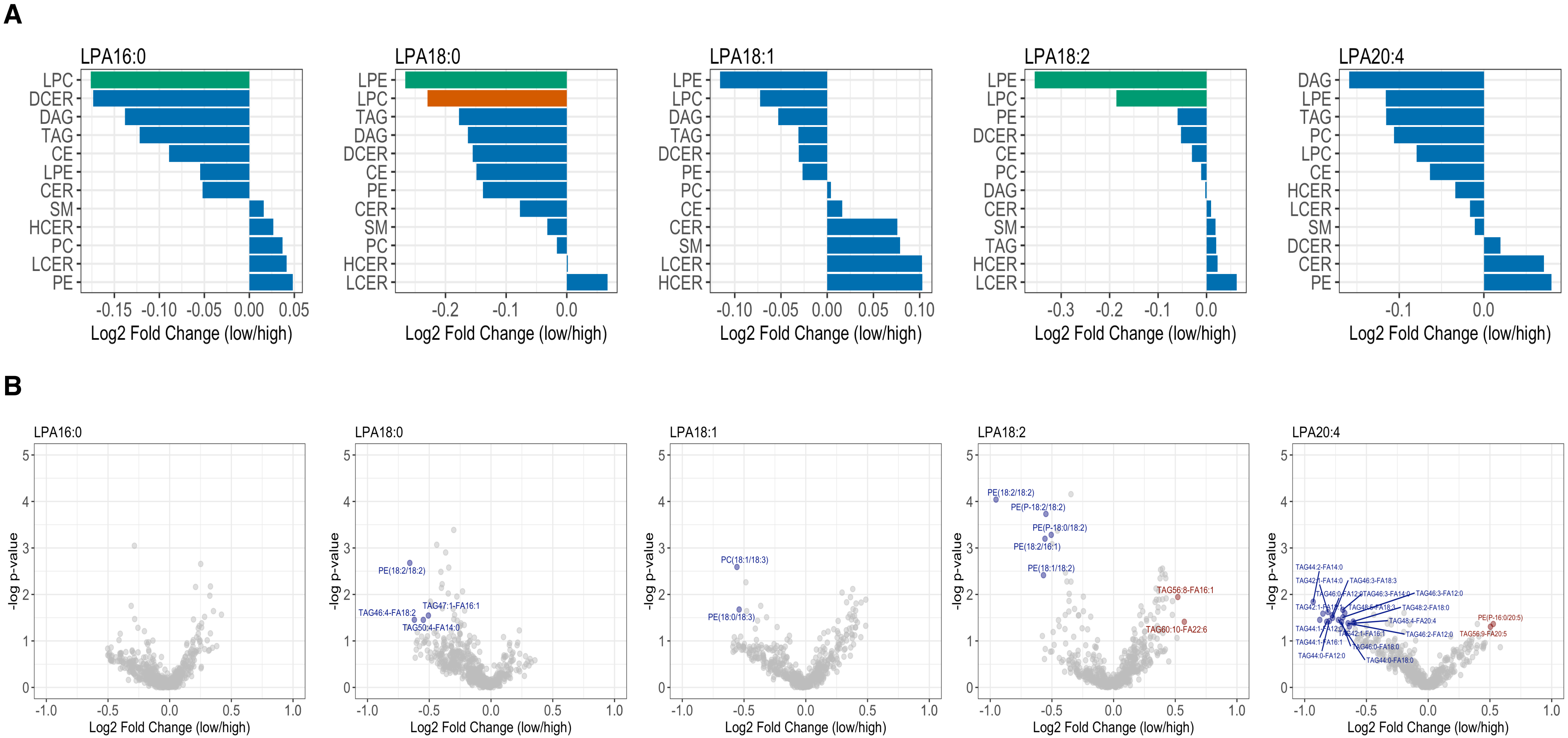

Figure S4. Differential lipid expression by baseline biomarker profile. (**A**) Profiling of 12 classes of lipids in LPA low versus high patients. The *x* axis denotes the average $\log_2$ (analyte abundance in low/analyte abundance in high); values less than 0 indicate a decrease, and values greater than 0 an increase, in low versus high patients. Green bars denote unadjusted p-value <0.05; orange bars denote false discovery rate <0.05. CE, cholesteryl esters; CER, ceramides; DAG, diacylglycerols; DCER, dihydroceramides; HCER, hexosylceramides; LCER, lactosylceramides; LPC, lysophosphatidylcholines; LPE, lysophosphatidylethanolamines; PC, phosphatidylcholines; PE, phosphatidylethanolamines; SM, sphingomyelins; TAG, triacylglycerols. (**B**) Volcano plots showing lipid species in LPA low versus high patients. The *x* axis denotes $\log_2$(analyte abundance in low/analyte abundance in high), and the *y* axis indicates the $-\log_{10}$(unadjusted p-value).

Colored circles denote unadjusted p-value<0.05; red circles denote lipid species with higher abundance in LPA-low compared to LPA-high (fold change>0.5); blue circles denote lipid species with lower abundance in LPA-low compared to LPA-high (fold change>0.5).
